# Photographic Analysis of a Low-Current, Vacuum Electric Arc Using an Ultrafast Camera

**DOI:** 10.3390/ma18030693

**Published:** 2025-02-05

**Authors:** Michał Lech, Paweł Węgierek

**Affiliations:** Department of Electrical Equipments and High Voltage Technology, Faculty of Electrical Engineering and Computer Science, Lublin University of Technology, Nadbystrzycka 38A, 20-618 Lublin, Poland; p.wegierek@pollub.pl

**Keywords:** vacuum arc, vacuum interrupter, microparticles, plasma, photographic analysis, arc flash, phases of vacuum arc, correlation of voltage and light emission

## Abstract

The main component of vacuum interrupters responsible for ensuring the correct flow of current is the contact system. In a vacuum environment, due to the higher values of the mean free path of electrons and particles in the contact gap, the material and condition of the contacts exert the greatest influence on the development of the arc discharge. To accurately analyze the phenomenon of discharge development in vacuum insulating systems, the authors conducted a time-lapse photographic analysis of a vacuum electric arc. For this purpose, they used a test setup comprising a discharge chamber, a vacuum pump set, a power and load assembly, an ultra-high-speed camera, and an oscilloscope with dedicated probes. The measurement process involved connecting the system, determining the power supply, load, and measurement parameters and subsequently performing contact opening operations while simultaneously recording the process using the oscilloscope and ultra-high-speed camera. An analysis of a low-current vacuum arc in a residual helium gas environment, with a pressure of *p* = 1.00 × 10^1^ Pa was carried out. Different phases of vacuum arc burning between electrodes in the discharge chamber were identified. In the stable phase, the arc voltage remained constant, while in the unstable phase, the arc voltage increased. The results of the time-lapse analysis were compared with the characteristics recorded by the oscilloscope, revealing a correlation between the increase in vacuum arc voltage and the intensity of flashes in the interelectrode space. The movement of microparticles ejected from the surface of the contacts—either reflecting or adhering to one of the electrodes—was observed. This analysis provides a deeper understanding of the processes involved in discharge formation and development under reduced pressure conditions. Understanding these mechanisms can support the design of vacuum interrupters, particularly in the selection of suitable contact materials and shapes.

## 1. Introduction

Contacts in vacuum interrupters are a critical element in ensuring proper operation by facilitating the flow of electric current in the circuit. Vacuum switching technology is widely utilized in electrical power switchgear, with applications including medium- and high-voltage circuit breakers, disconnectors, and contactors. For this type of insulating medium, the material properties of the vacuum interrupter contacts are crucial during arc quenching and the recovery of electrical strength [1]. This is primarily due to the fact that, in a high-vacuum environment, the mean free path of electrons and residual gas molecules exceeds the contact gap, allowing them to traverse the gap without collisions. Additionally, the diameter of the residual gas molecules plays an important role, as larger molecules result in a shorter mean free path.

The mean free path of gas molecules *L*_m_ can be calculated using the following equation [2,3,4]:

$$L_{m}=3.11\cdot 10^{-24}\frac{T}{pd_{m}^{2}}$$

where *T*—absolute temperature of the gas [K], *p*—gas pressure [Pa], and *d*_m_—diameter of the gas molecule [m].

Similarly, the mean free path of electrons *L*_e_ in the gas can be determined using [2,3,4]:

$$L_{e}=1.76\cdot 10^{-23}\frac{T}{pd_{e}^{2}}$$

When disconnecting an electrical circuit where the current is at least equal to the critical value (*I*_lim_) and the voltage between the contacts exceeds the critical voltage (*U*_lim_), an arc is initiated. These threshold values depend on the contact material. Arc ignition always results from the release of metal vapors from the contact surfaces.

Long-term research on the arc burning process in switching devices has shown that the electric arc formed between separating contacts occurs in the metal vapor produced by the molten metal bridge. For this reason, such arcs are referred to as metallic arcs [5]. In a vacuum, an arc is similarly formed through the vaporization of metal from the contacts. There are three distinct types of vacuum arcs [1]:

- Diffusion vacuum arc (currents ≤ 6 kA),
- Transient vacuum arc (currents > 6 kA and ≤10 kA),
- Column vacuum arc (currents > 10 kA).

### 1.1. Diffusion Vacuum Arc

**Figure 1:**
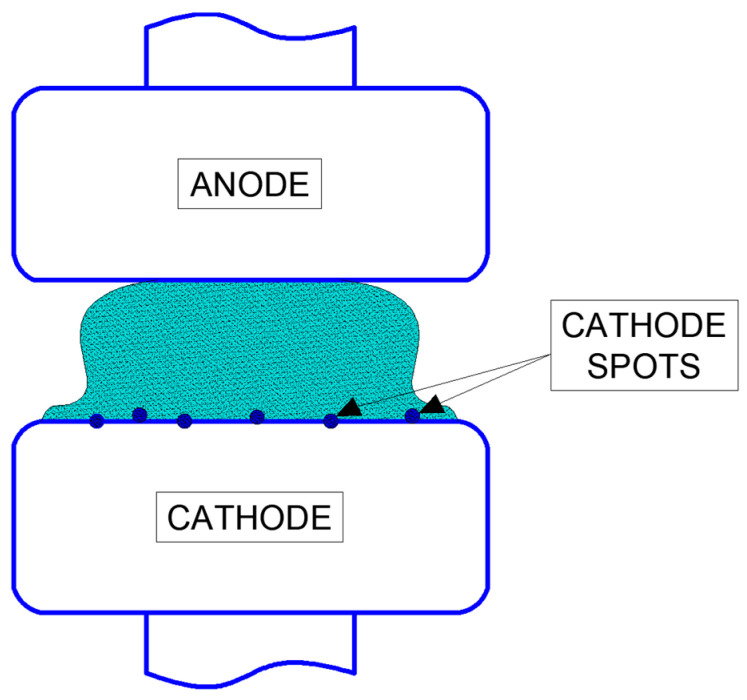
Diffusion vacuum arc.

An example of a diffusion vacuum arc is illustrated in Figure 1. The cathode surface exhibits bright regions known as cathode spots, which move randomly and repel one another, changing their positions in the opposite direction. Between the apex of the cathode spot and the anode lies a diffuse, neutral plasma that facilitates current flow in the circuit with minimal voltage drop. The anode plays a passive role, collecting electrons across its surface.

It is generally accepted that there are two types of cathode spots: type I and type II. Type I cathode spots appear on contacts covered with oxide layers or other impurities. Even thick oxide layers (several μm) are removed by cathode spots moving across the surface [6]. Contact surfaces in vacuum interrupters are cleaned during both the manufacturing process and each switching operation, thanks to the action of the vacuum arc. Therefore, designers of vacuum interrupters must consider only type II cathode spots. Typical characteristics of this type are presented in Table 1.

**Figure 2:**
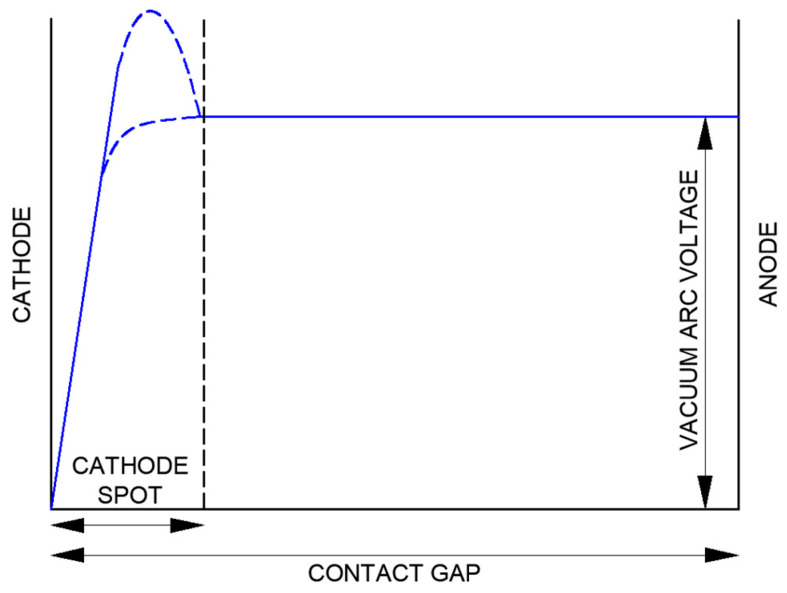
Arc voltage in a distributed vacuum arc with closely spaced contacts (own development based on [1]).

At first glance, the plasma above the cathode spot in the interelectrode space appears diffuse, faintly luminous, and neutral, serving only to conduct arc current from the anode to the cathode. In this arrangement, the anode acts as a passive electron collector. However, a closer examination reveals that the physical processes occurring in the plasma significantly influence the nature of the discharge. For small contact gaps of 2 mm or less, almost the entire voltage drop is concentrated at the cathode spot, with virtually none occurring within the plasma itself. This phenomenon is illustrated in Figure 2. For large-diameter contacts, typical of vacuum interrupters, the total voltage of the dissipated vacuum arc typically ranges from approximately 20 V to 45 V, with currents up to 3150 A and a standard contact gap ranging from 8 mm to 20 mm.

In the case of a vacuum electric arc, the entire space between the contacts is filled with metal vapors emitted from cathodic sites. The process of contact material erosion has been extensively studied over the years. Table 2 presents erosion rates for materials commonly used in vacuum interrupter contact systems. These rates are defined as “grams per coulomb”, representing the amount of material the cathode loses relative to the product of the current and the arc discharge duration.

Studies [7,8] have shown that most material loss results from the emission of contact material particles, likely due to the rapid formation and movement of cathode spots across the cathode surface [9,10]. The emitted metal particles, ranging in diameter from fractions of a micrometer to tens of micrometers, travel at velocities of 10 to 800 m/s and reach temperatures higher than the melting point but below the boiling point of the cathode material

During an arc discharge, most metallic particles are ejected horizontally along the cathode plane. In vacuum interrupters, these particles are deposited on the ion trap surrounding the contact system.

The angular distribution of ions above the cathode points (plasma cloud) in low-current vacuum arcs (with two or three cathode points) follows a cosine distribution [11]. At higher currents (12–20 cathode points), the distribution becomes flatter. For simplicity, the plasma cloud from each cathode point can be assumed to extend within a cone of approximately 70 degrees, perpendicular to the cathode surface [12].

During vacuum arc dissipation, multiple cathode points are typically present in vacuum interrupters when the contacts disconnect. Each cathode point generates a separate plasma cloud, and if the density of cathode points is sufficiently high (corresponding to a high current for a given contact radius), these plasma clouds overlap before reaching the anode. As the current increases, the cathode point density grows, leading to an overlapping plasma region near the cathode.

As shown in Table 3, cathode spots remain distributed across the cathode surface. However, instead of separate plasma clouds, an overlapping plasma region extends to the anode. Within this region, plasma collisions intensify as the density increases, giving rise to elastic and inelastic collisions that influence local ionization processes.

Some microparticles detached from the cathode spots are vaporized within the plasma cloud. As plasma density further increases and collisional phenomena intensify, an azimuthal magnetic field *B* can be induced in the plasma by the current flow. This phenomenon may result in the “compression” of the arc column, as illustrated in Table 3, leading to an increase in vacuum arc voltage.

### 1.2. Column Vacuum Arc

**Figure 3:**
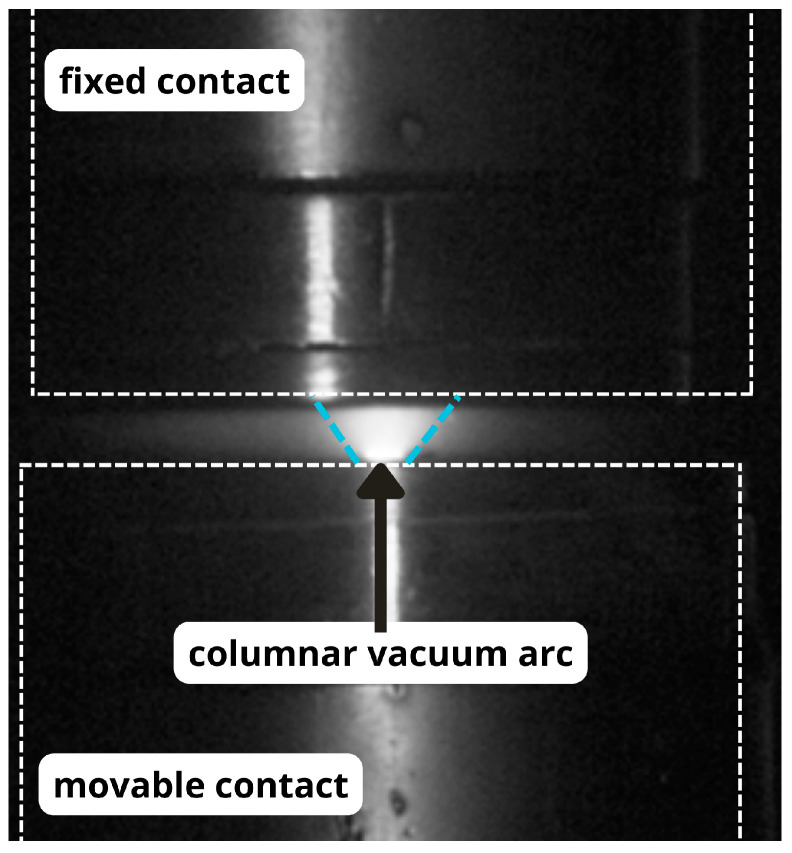
Column vacuum arc.

The column vacuum arc plays a critical role in the development of vacuum interrupters designed to extinguish high short-circuit currents. Understanding the mechanisms of its formation and control is essential. When contacts in a vacuum separate under high current, there is a transition from a molten metal bridge to a columnar bridge arc. At sufficiently high currents, the energy delivered to the contact surface balances the gradual expansion of the columnar arc radius and the emission of material into the surrounding vacuum. Consequently, the bridging column transforms into a high-pressure columnar arc, exhibiting properties similar to an arc in air at atmospheric pressure. An image of the columnar vacuum arc captured with a camera is shown in Figure 3.

**Figure 4:**
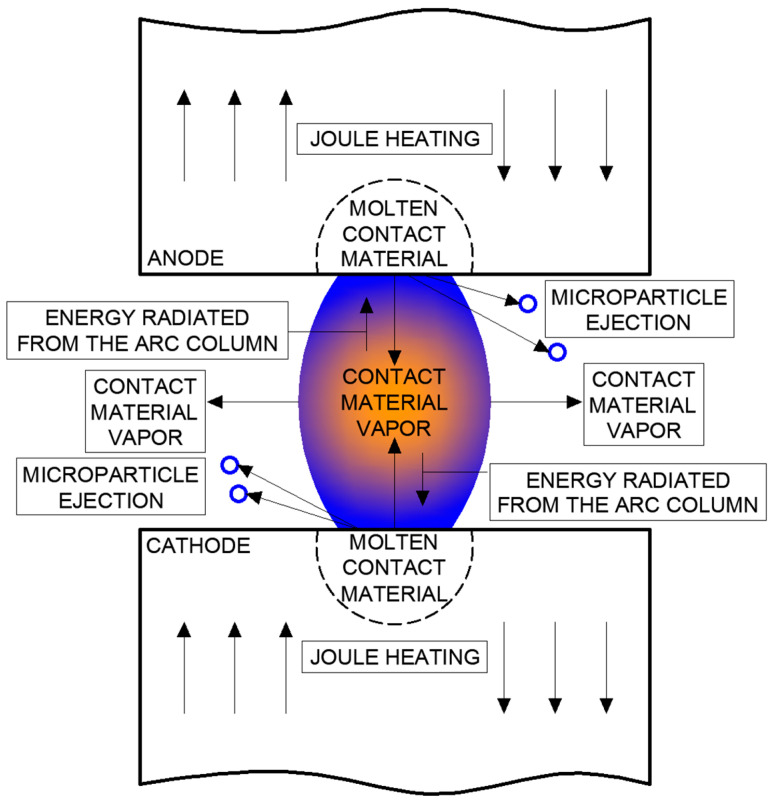
Energy balance for column vacuum arc (own development based on [1]).

Figure 4 summarizes the energy balance at the contacts for this type of vacuum arc. Detailed studies of stationary columnar vacuum arc formation between disconnecting contacts are complex, and few models exist to describe this phenomenon [1].

High-current vacuum arc behavior has been analyzed in various studies [1,13]. Using photographic analysis, researchers have described the behavior of the vacuum arc in vacuum interrupters. This process is typically recorded using high-speed cameras. By measuring the contact gap in each frame, researchers can track arc duration and correlate the onset of the arc with voltage changes. The first visible bright spot indicates the moment of arc ignition, allowing for the association of individual frames with arc-related phenomena, such as voltage fluctuations, current changes, and contact gap evolution.

**Figure 5:**
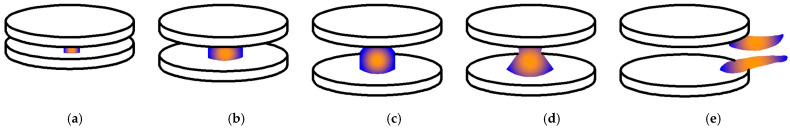
Modes of the formation of high-current vacuum arc for butt contacts (own development): (**a**) the bridge column arc, (**b**) the diffuse column arc, (**c**) the constricted column vacuum arc, (**d**) the plasma jet column vacuum arc, and (**e**) the anode and cathode jet vacuum arc.

Figure 5 illustrates the appearance of a high-current vacuum arc between opening flat contacts.

The behavior of the electric arc between the contacts is described as follows:

(a) It occurs immediately after the molten metal bridge breaks.
(b) It develops from a bridged column arc at currents between approximately 6 kA and 15 kA.
(c) It transitions from a bridged column arc to a distributed column arc for currents exceeding 15 kA.
(d) It resembles a constricted column arc, with a narrowed central area and an arc column width at the cathode larger than at the anode.
(e) At currents exceeding 20 kA and as the contact gap widens, the arc column shifts to the edge of the contacts.

The study of physical phenomena in vacuum switching apparatus has garnered significant attention in recent years. One important research focus is the analysis of microparticles generated during vacuum arc burning [14,15,16,17,18,19,20]. These studies examine the dynamic behavior of plasma–particle interactions, such as oscillation and levitation, and the impact of microparticles on vacuum interrupters’ breakdown and insulation capabilities. This body of work includes both numerical simulations and experimental research on microparticle formation, erosion, and discharge mechanisms. Metallic particles can amplify local electric fields and electron emissions, increasing the likelihood of late breakdowns. Contact material properties, such as the chromium content in CuCr alloys, influence particle generation dynamics, with higher chromium content reducing particle formation risks. Even after the current ceases, particle motion persists in the system, potentially causing late breakdowns. This research highlights strategies to mitigate particle-related issues, such as optimizing electrode materials or modifying operating parameters, to improve vacuum interrupter performance and reliability.

Studies [21,22,23] have also explored vacuum arc parameters, including current, voltage, and energy, through theoretical and experimental approaches. Accurate measurements and analyses of these parameters are essential for understanding and enhancing vacuum circuit breaker performance. This research underpins advanced diagnostic methods and mathematical models that improve arc dynamics understanding and optimize equipment design and operating parameters.

Other studies have investigated the influence of magnetic fields on vacuum arc characteristics and behavior [24,25,26]. Research in this area focuses on how transverse magnetic fields (TMFs), axial magnetic fields (AMFs), and external transverse magnetic fields (ETMFs) affect arc stability, cathode erosion, and heat distribution. Magnetic fields are critical for controlling vacuum arcs and minimizing local damage, with advanced contact designs (e.g., incorporating TMFs and AMFs) significantly improving performance.

Research on contact materials has also been a major focus [27,28,29,30,31]. These studies, using experimental and simulation approaches, aim to identify materials that withstand high currents and temperatures while minimizing erosion and welding. Advanced alloys, such as CuCr, have been highlighted for their superior performance. Technologies such as AMFs have shown potential for further improving equipment durability and efficiency.

The purpose of this paper is to analyze the dynamics of low-current vacuum arc development under low-pressure conditions using an ultrafast camera to better understand the mechanisms of vacuum arc formation and development. The study seeks to identify key factors affecting arc stability, such as microparticle motion, electric field inhomogeneities, and voltage changes, while correlating observed optical effects with the discharge’s electrical characteristics.

## 2. Test Stand and Measurement Methodology

**Figure 6:**
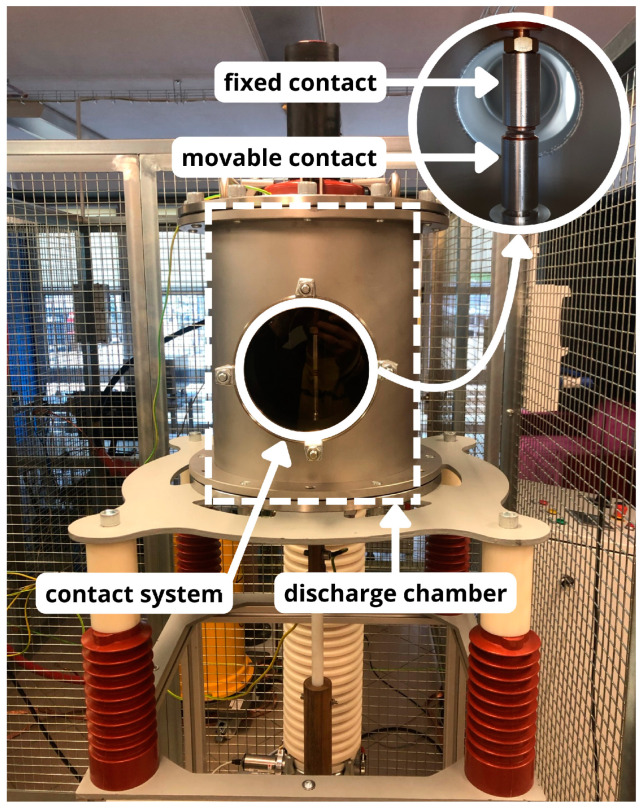
Discharge chamber with visible contact system inside.

The test stand used in this study is part of the equipment in the Faculty Laboratory of Switching and Distribution Equipment at the Faculty of Electrical Engineering and Computer Science, Lublin University of Technology. The core of the laboratory setup is a demountable vacuum chamber equipped with a contact system mounted inside. This setup enables precise measurement of arc parameters during switching operations. The chamber is operated by an electromagnetic drive, adapted from a medium-voltage circuit breaker, which works in conjunction with a dedicated bay controller. The bay controller allows for precise opening and closing of the contact system. The vacuum chamber is equipped with special sight glasses at the contact height, enabling observation of the contact system during testing. A view of the discharge chamber with the mounted contact system is shown in Figure 6.

**Figure 7:**
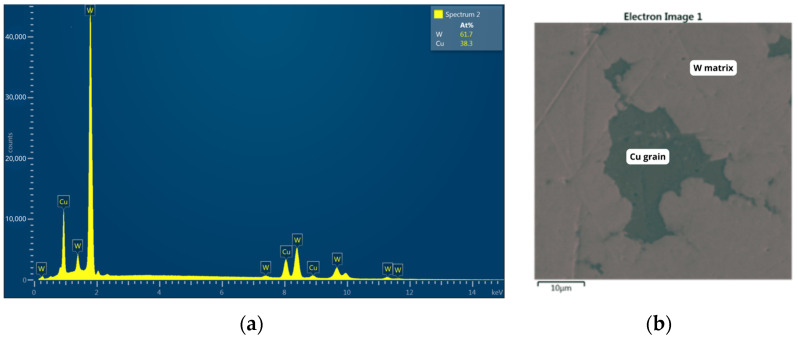
(**a**) Characteristics of the material of the contact pads mounted in the discharge chamber and (**b**) photo taken with an electron microscope.

The test object within the laboratory setup is a contact system terminated with pads made of WCu (tungsten–copper) material. Figure 7 provides both an electron microscope image and a summary of the material’s characteristics.

SEM imaging was obtained using a scanning electron microscope system working in a high vacuum regime equipped with a scintillator YAG secondary electron detector. One can see a highly irregular Cu grain of approximately 40 um x 30 um in size, with several axon-shaped appendices of length ~10 um in the W matrix. EDS microanalysis performed using an EDS advanced system with a 10 mm^2^ X-act SSD silicon detector confirmed the general composition of the material, based on several characteristic X-ray lines of W and Cu, shown in EDS spectra. Any important impurities (with a concentration higher than 0.1 weight %) were not detected. However, one should have in mind that composition determined by an automated system should be taken cum grano salis as it may vary with the position of the optical axis in the case of such highly inhomogeneous (in three dimensions) material.

The discharge chamber cooperates with a vacuum pump system, which includes a vacuum pre-pump and a vacuum main pump. The pre-pump was developed as a rotary oil vane pump and had a pumping rate of 8.5 m^3^/h. The main vacuum pump is a turbomolecular pump with magnetic impeller suspension and an integrated controller. Its pumping rate is 365 l/s for nitrogen, 280 l/s for helium, and 200 l/s for hydrogen, while the final pressure is <10^−8^ mbar. The pressure in the system is monitored by two vacuum gauges that measure it at different points in the pumping channel. The vacuum gauges have two measurement channels and a display range of 10^−10^ ÷ 2000 mbar. The stand also allows the connection of technical gases to the system, which facilitates the introduction of residual gases other than air into the interstitial space.

The following devices were used to record and measure the parameters of switching operations associated with arc phenomena in the discharge chamber:

- A 1 GHz oscilloscope with probes to record the current and voltage of the electric arc generated between contacts during switching operations;
- An ultra-high-speed monochrome camera with a throughput of 7 Gpx/s, a maximum resolution of 1280 × 800 pixels, and a speed of up to 7400 frames per second at full resolution. The camera reaches a maximum speed of 1,000,000 frames per second at 64 × 8 pixels, with a minimum exposure time of 1 µs. Mounted on an adjustable tripod, the camera, together with a contact illumination lamp, allows for the recording of the phenomena of arc breakdown and the process of arc burning between contacts.

**Figure 8:**
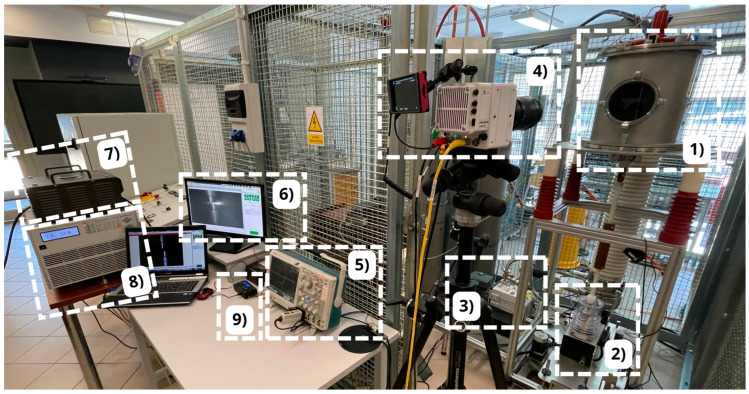
View of the laboratory station used to test switching operations: 1—discharge chamber with the contact system, 2—electromagnetic drive, 3—pre-pump, 4—ultrafast camera, 5—oscilloscope, 6—computer workstation, 7—autotransformer, 8—electronic load cell, and 9—delay controller and “zero-crossing” detector.

The power supply for the bench is provided by an autotransformer, which supplies voltage to the discharge chamber under test. The system’s load is an electronic load cell rated at 3.6 kW, with an adjustable voltage range from 50 V to 350 V, continuous current up to 36 A, and peak current up to 108 A. To control the timing of contact opening, a delay time generator is used in conjunction with a “zero-crossing” detector. This allows precise synchronization of contact opening with the current waveform. A complete view of the test bench, designed for analyzing switching operations in a vacuum discharge chamber, is shown in Figure 8.

**Figure 9:**
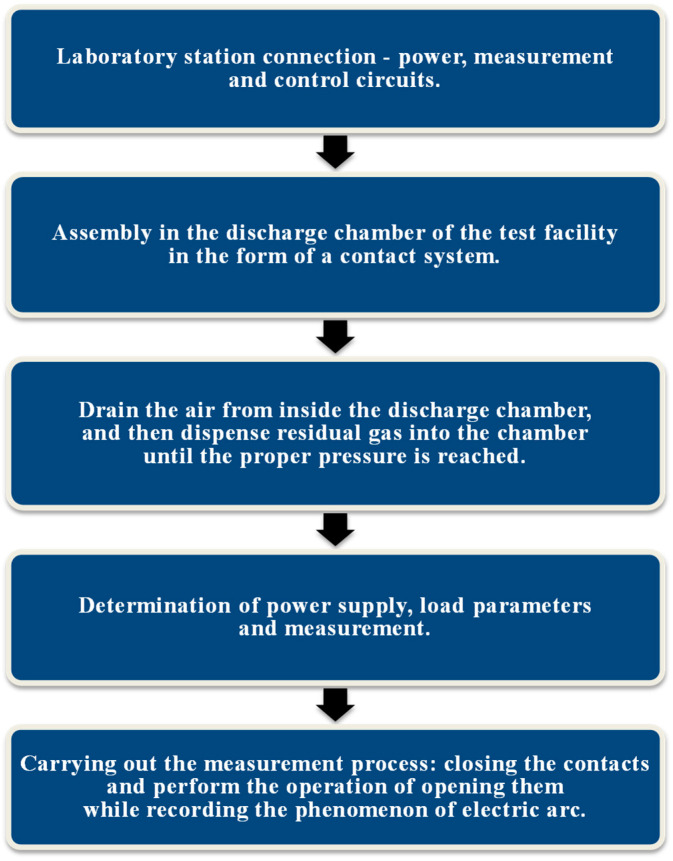
Block diagram showing the methodology for carrying out the analysis of the phenomenon of low-current vacuum electric arc.

**Figure 10:**
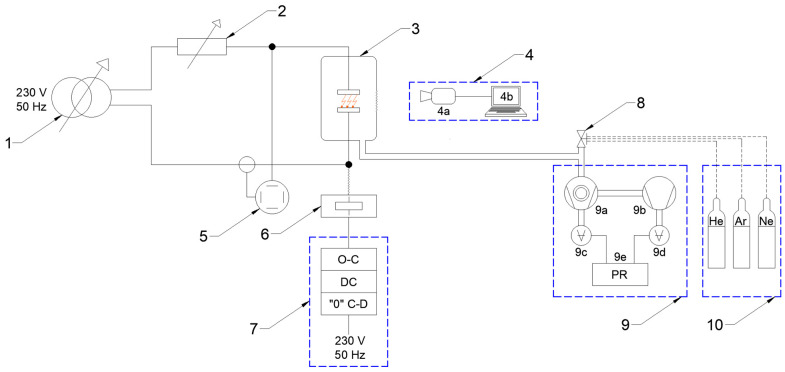
Schematic of the laboratory station designed to study the arc phenomenon: 1—autotransformer, 2—electronic load cell, 3—discharge chamber with the contact system, 4—set for photographic analysis (a—ultrafast camera and b—computer workstation), 5—oscilloscope, 6—electromagnetic drive, 7—control panel with the option to open and close the contacts of the chamber, delay controller and “zero-crossing” detector, 8—vacuum manual valve, 9—vacuum set (a—turbomolecular vacuum pump, b—pre-pump, c, d—vacuum gauges, and e—pressure regulator), and 10—set of technical gases.

Once the system was properly connected, the key issue was to assemble it in the discharge chamber of the test facility in the form of a contact system. Using a vacuum kit, air was pumped out of the interior of the discharge chamber, and then, using appropriate valves, residual gas in the form of helium was dispensed into the chamber. After the vacuum chamber was pressurized, the supply and load parameters were determined. Then, the measurement system—an oscilloscope and an ultrafast camera—was put into operation. In order to correctly record the vacuum arc phenomenon, it was crucial to properly set up the camera and illumination and determine the appropriate camera parameters. Then, the contact system was closed, the load was attached, and the contact system was opened. Just before opening the contacts, it was necessary to start recording, which was saved on a computer disk for later analysis. A block diagram showing the methodology for performing measurements of selected parameters of an electric arc in a vacuum discharge chamber is shown in Figure 9, while an electrical diagram is shown in Figure 10.

## 3. Photographic Analysis of a Vacuum Electric Arc

**Figure 11:**
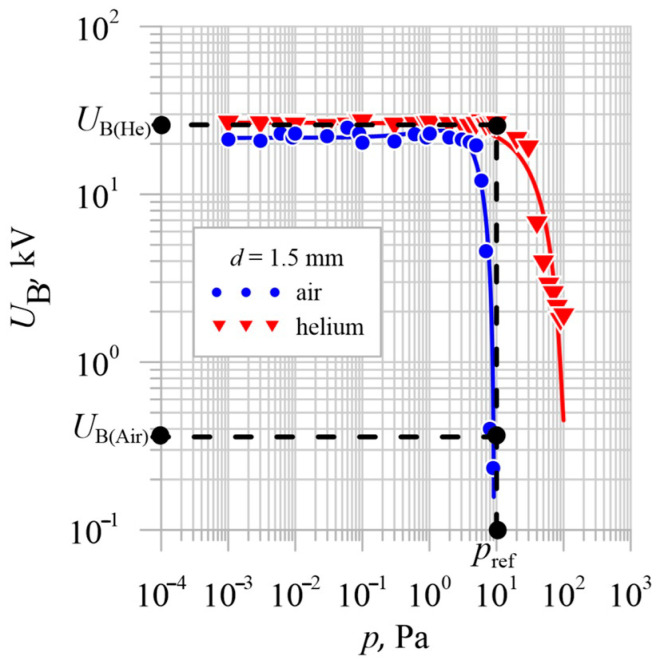
Characteristics of *U*_B_ = f(*p*) for a vacuum interrupter with vacuum generated based on air and helium for a contact gap of *d* = 1.5 mm [3].

In the previously published works by the authors [2,3], it was demonstrated that using selected noble gases, such as neon and helium, to create vacuum conditions in interrupters allows for an increase in the operating pressure of these components while maintaining electrical strength comparable to a vacuum based on air. The most promising results were obtained with helium as the insulating medium, as shown in Figure 11. The relative shift in the characteristics of *U*_B_ = f(*p*) for air and helium can be attributed to helium’s high first ionization energy (2370 kJ/mol), which surpasses that of other residual gases used in the study, such as air, neon, and argon. Furthermore, helium is an environmentally benign medium, aligning with the latest guidelines for insulating gases in electrical switching equipment [5].

Given the rated operating pressure of vacuum interrupters, approximately 10^−3^ Pa, increasing the operating pressure reduces the likelihood of potential unsealing of the interrupter and thus the risk of transferring potential to the opposite pole of the switching apparatus.

Considering the results shown in Figure 11, the authors decided to conduct time-lapse recordings of the contact opening process in a residual gas environment using helium at a pressure of *p*_ref_ = 1.00 × 10^1^ Pa. At this pressure, vacuum interrupters with air as the residual gas lose their insulating capacity, while in the case of helium, the electrical strength remains constant and safe.

**Figure 12:**
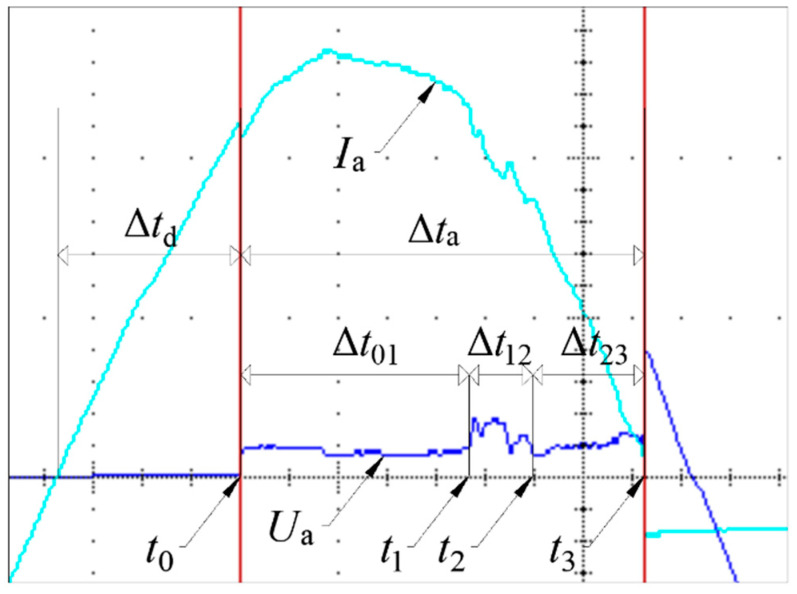
Characteristics of current and voltage during the arc burning process at a pressure equal to *p*_ref_ = 1.00 × 10^1^ Pa: *I*_a_—the value of the electric current flowing through the contact system, *U*_a_—the voltage of the vacuum arc, Δ*t*_d_—the delay time after which the arc ignited, Δ*t*_a_—the burning time of the electric arc, and *t*_0_—the moment of ignition of the vacuum arc.

Using an oscilloscope and a high-speed camera, the vacuum arc burning process was recorded under a supply voltage of 230 V and a current of 10 A. Figure 12 shows an example of the current flowing in the circuit and the voltage of the vacuum arc at a pressure of *p*_ref_ = 1.00 × 10^1^ Pa in the discharge chamber filled with helium.

Based on the analysis of the figure below, three time intervals of the arc burning process can be distinguished. In the Δ*t*_01_ interval of about 3.738 ms, the arc burns stably, while in the Δ*t*_12_ interval of about 1.040 ms, the arc enters an unstable phase. In the final phase Δ*t*_23_ of about 1.830 ms, the arc burns stably again until it goes out at *t*_3_. The total arc burning time in this case was about 6.608 ms. The same phenomenon was analyzed photographically. Table 4 summarizes the parameters used for testing the arc with the ultrafast camera.

**Figure 13:**
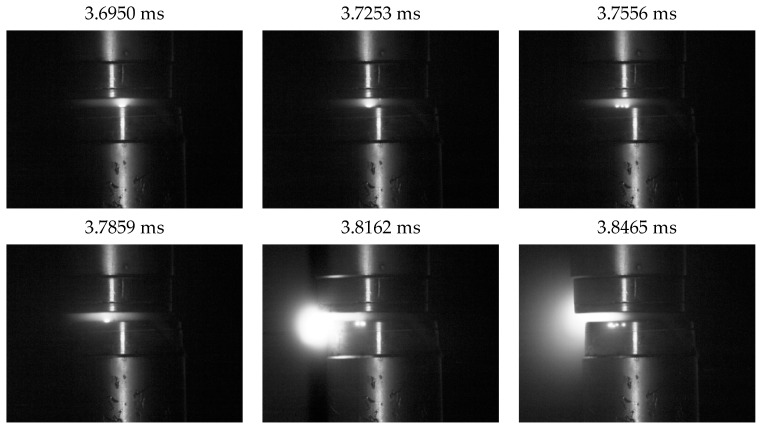

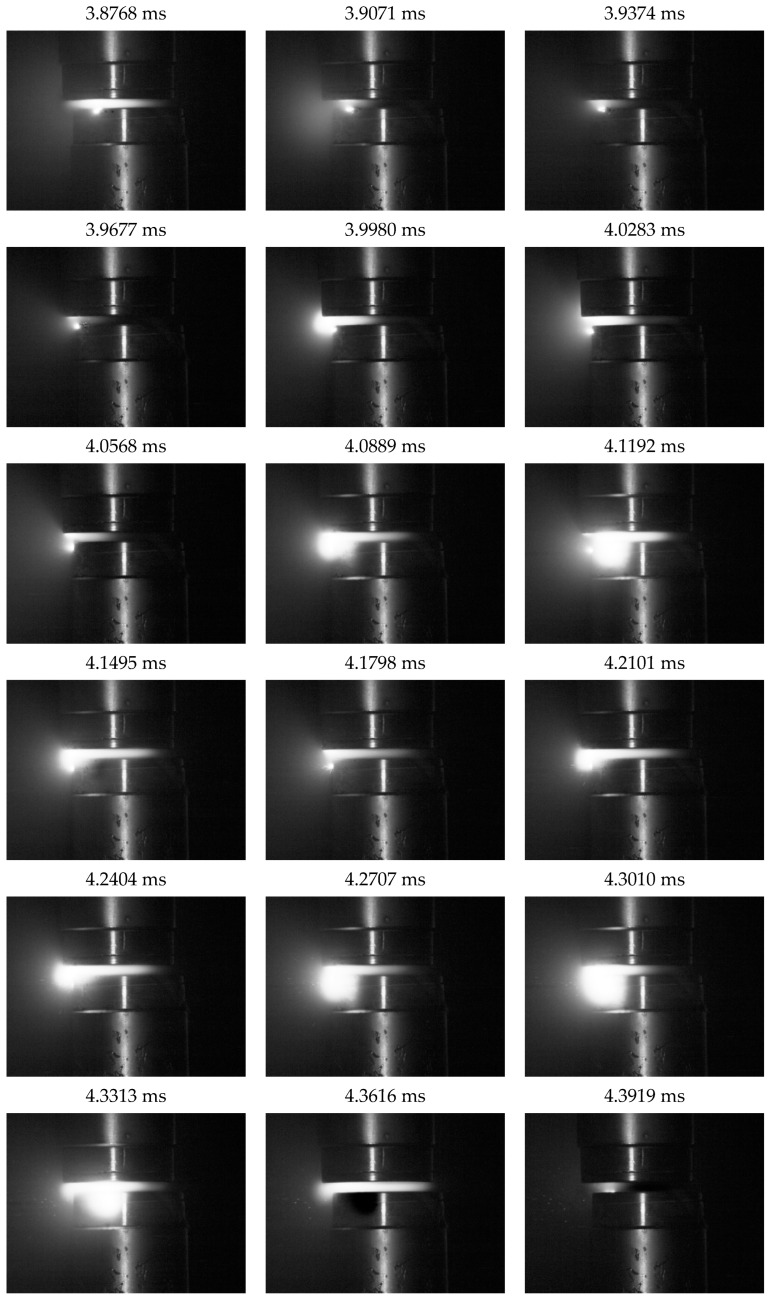

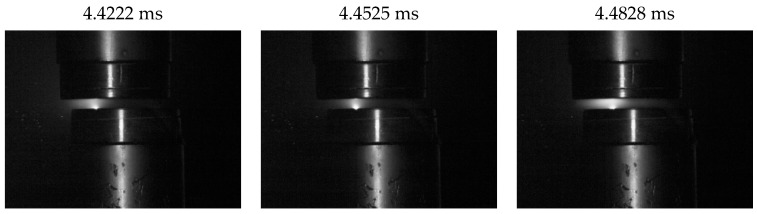
Time-lapse analysis of a fragment of arc burning in the unstable phase.

Figure 13 provides a time-lapse analysis of a selected part of the vacuum arc burning process, corresponding to the characteristics shown in Figure 12. At t = 3.7859 ms, a significant flash due to arc burning between the contacts becomes visible in the contact gap. This corresponds to the voltage rise of the vacuum arc at t_1_ in Figure 12. Subsequent frames clearly show repeated intense flashes, which diminish after approximately 0.7 ms. This reduction is associated with a voltage drop in the burning arc and the transition to a stable burning phase.

As for the differences in the shape of the arc for the different phases of its combustion, they can be divided as follows.

In the stable phase, the arc usually has a uniform columnar shape, which is the result of stabilized current flow between the electrodes. Stable arc burning conditions are associated with uniform electric field distribution and the absence of dynamic disturbances in the plasma. In the analyzed photographic data from this phase, a clear and relatively uniform glowing of the arc over the entire length of the interstitial space is visible.

In the unstable phase, dynamic changes in the arc shape were observed. In time-lapse images, fragmentation of the arc can be seen, and in some cases the formation of side branches or separation into several columns. These changes are probably the result of local fluctuations in plasma density, temperature, and electric field inhomogeneities. A characteristic phenomenon in this phase is the increased intensity of flashes, which may be related to the emission of microparticles and their interaction with the plasma. Instability leads to an irregular arc shape, which is more stretched or irregular compared to the stable phase.

The transition between stable and unstable phases is of particular interest. The images recorded cases where the arc went from a uniform columnar shape to a stretched, wavy structure, indicating increasing hydrodynamic and magnetic instabilities. In this phase, the shape of the arc can be strongly influenced by local conditions, such as the presence of residual gas particles or the drift of electrode material.

**Figure 14:**
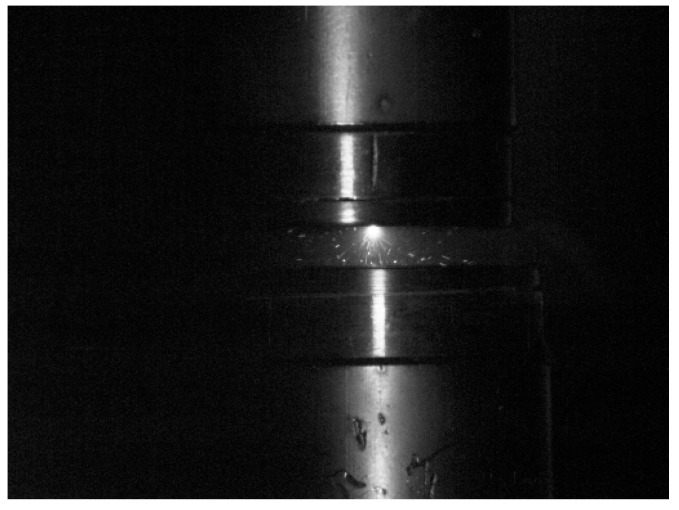
Vacuum electric arc with the arc column at the upper stationary contact phase.

**Figure 15:**
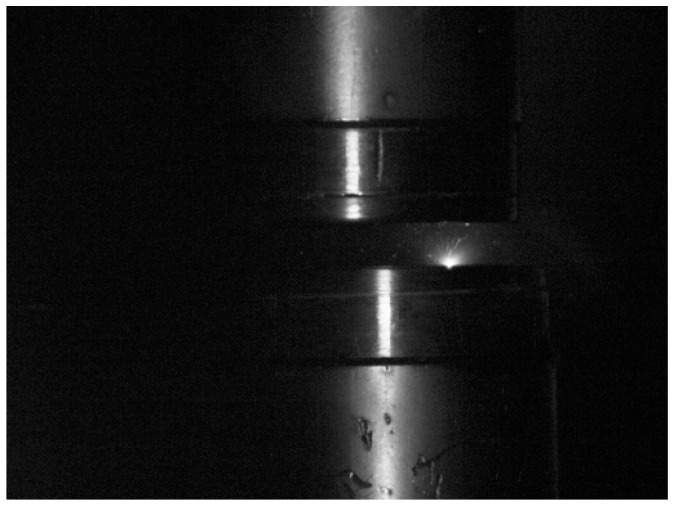
Vacuum electric arc with the arc column at the lower moving contact phase.

The photographic analysis revealed that the low-current vacuum arc typically burns in one of three characteristic ways. Most commonly, the arc is confined to a single arc column, which is usually held at the stationary contact (Figure 14), although cases were observed where the column was held at the moving contact (Figure 15).

**Figure 16:**
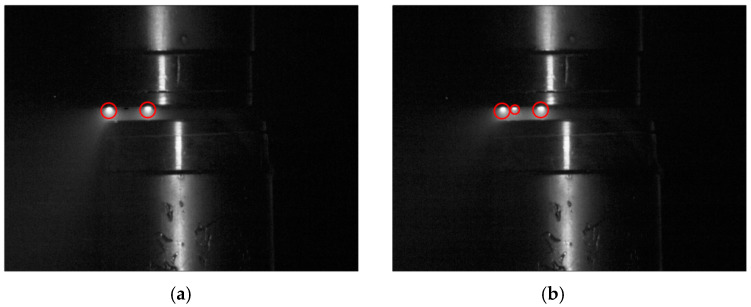
Vacuum electric arc with two (**a**) and three (**b**) arc columns visible.

In some instances, the arc is divided into multiple arc columns, as shown in Figure 16. From the perspective of minimizing contact surface degradation, this is a favorable phenomenon, as it reduces the energy concentration at any one point.

In vacuum quench systems, the distribution of the electric field in the interstice space can be inhomogeneous due to irregularities in the contact surfaces, local damage or the presence of microparticles knocked out of the electrode surfaces. These inhomogeneities lead to local electric field maxima, which can initiate separate plasma channels. Each such channel can evolve into an independent arc column, especially when local conditions favor the development of a discharge.

The splitting of the arc into several columns can also be caused by local changes in plasma parameters, such as temperature, ion and electron density, or the concentration of residual gas molecules. In a vacuum environment with a small amount of helium, areas of different plasma densities can appear due to dynamic interactions between ions and microparticles. The movement of microparticles in the interstitial space, which we observed in the photographic analysis, may further contribute to local cooling or heating of the plasma, which promotes the formation of independent arc channels.

Moreover, when current flows through contacts under vacuum conditions, micro- and nanostructures on the contact surface can generate local discharges. These discharges can lead to the emission of particles or plasma that form local conduction paths, acting as nuclei for independent arc columns.

**Figure 17:**
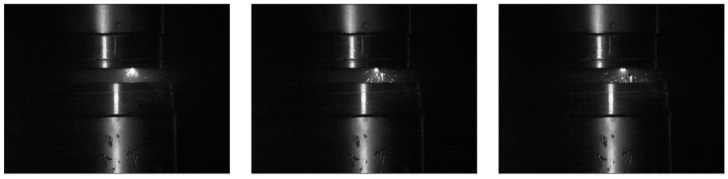
Phenomenon of microparticle detachment from the contact surface of an upper, stationary contact.

**Figure 18:**
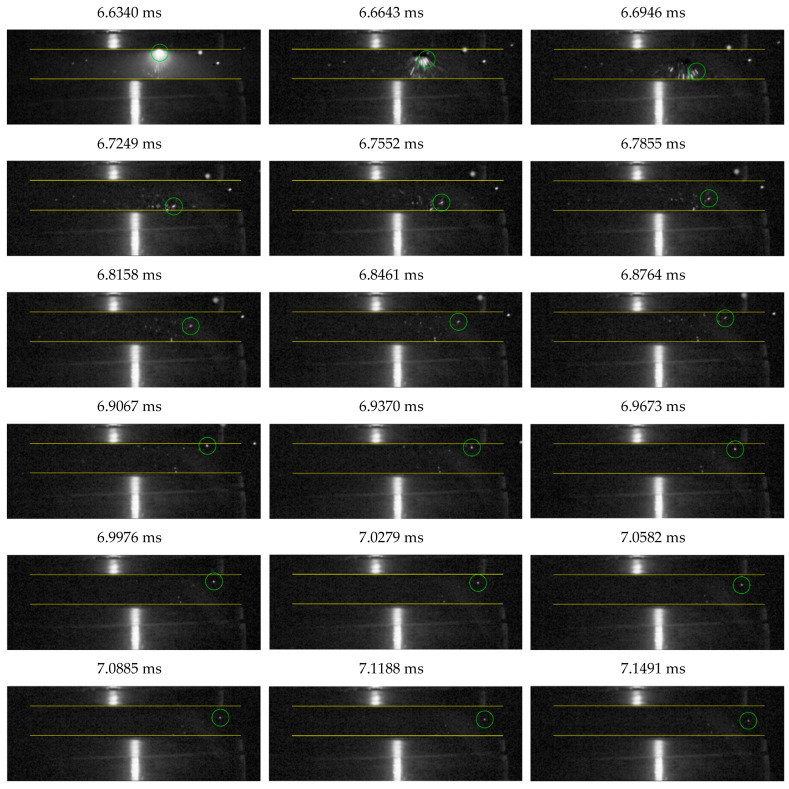

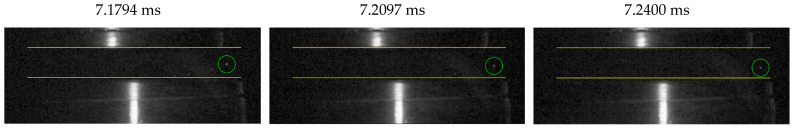
Time-lapse analysis of the motion of a microparticle bouncing off electrodes in a vacuum discharge chamber.

The movement of microparticles detaching from the contact surfaces was analyzed, as shown in Figure 17. Microparticles were observed to move in two ways: bouncing off the electrodes or attaching to one of them. Figure 18 illustrates the detailed movement of a single microparticle.

**Figure 19:**
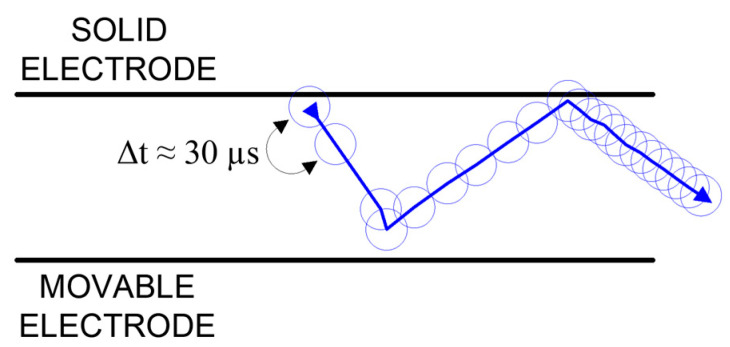
Movement of a microparticle bouncing off contacts.

Based on time-lapse analysis, each position of the microparticle bouncing off the contacts was transferred to a single drawing with the electrodes (contacts) marked. In this way, the path traveled by the microparticle bouncing off the contacts was mapped (Figure 19).

Based on the figure above, three phases of microparticle movement can be distinguished. In the first phase, the microparticle detachment of the stationary contact moves toward the moving contact. In the second phase, it bounces off the moving contact and returns toward the stationary contact. In the last phase, it bounces again from the stationary contact and is detached into the space of the discharge chamber. It should be noted that in the first phase, the microparticle moves at the highest speed, as the microparticle has traveled the longest distance between measurements, with a time of about 30 µs between them. In the second phase, a decrease in the speed of the microparticle is apparent, and in the last phase, the microparticle moves at the lowest speed. This is caused by the loss of kinetic energy after each successive collision with the contact.

## 4. Conclusions

This article presents a test stand and methodology for analyzing vacuum electric arcs, utilizing, among other tools, an ultrafast photographic camera that enables time-lapse recording of this phenomenon. The research focused on arcs occurring in vacuum interrupters used in electrical power-switching equipment. A series of time-lapse photographs were taken, and the current and voltage waveforms of the arc were recorded under a supply voltage of 230 V, with a current of 10 A flowing through the contacts of the discharge chamber. The discharge chamber was filled with residual gas (helium) at a pressure of *p*_ref_ = 1.00 × 10^1^ Pa. Based on the conducted tests, the following conclusions were drawn:

- In most of the analyzed cases, the electric arc burned stably, maintaining a constant voltage value.
- In the majority of cases, a single arc column was observed moving between the contacts.
- In some instances, the vacuum arc burning process exhibited stable and unstable burning phases, with the unstable phase characterized by an increase in arc voltage.
- The movement of microparticles between the contacts of the vacuum chamber can be categorized as either bouncing off the contacts or attaching to one of them.
- The use of an ultrafast camera enabled precise tracking of the movement of a selected microparticle, as illustrated in a specific example.

The photographic analysis of a low-current vacuum electric arc using an ultrafast camera made it possible to identify key stages in the development of the discharge and to establish a relationship between the arc voltage and its dynamic behavior in the interstitial space. The results obtained are of significant practical importance and may find applications in the following areas:

- Design of vacuum interrupters:

Understanding the mechanisms of arc development in a vacuum environment and identifying the factors affecting discharge stability can aid in optimizing the design of vacuum extinguishing chambers used in medium- and high-voltage distribution equipment. The study highlights the need to consider the effects of microparticles and electric field inhomogeneities on arc behavior, which may lead to the selection of more resilient contact materials or changes in contact geometry to minimize arc instability.

- Diagnostics and monitoring of electrical contacts:

The observed relationships between increases in arc voltage and the intensity of flashes in the interstitial space could serve as the foundation for developing diagnostic systems for vacuum devices. Using high-speed recording cameras or optical detectors could enable the early detection of contact degradation and the assessment of their technical condition, thus enhancing the operational reliability of the equipment.

- Development of low-pressure arc quenching technology:

The results of this study can be adapted for other applications where managing electrical discharges under reduced pressure is critical. These include vacuum chambers in research laboratories, high-vacuum devices in the semiconductor industry, and insulation systems for space technologies.

- Numerical simulations of plasma processes:

The experimental data obtained on the arc’s shape, development dynamics, and the effect of gas conditions on arc combustion provide a valuable reference for validating numerical models that simulate plasma behavior in vacuum systems. This could lead to the design of more precise and efficient electrical devices without the need for costly large-scale experiments.

The results of this study contribute to a deeper understanding of the mechanisms underlying arc discharge development in vacuum environments. They form a solid foundation for designing more efficient and reliable high-voltage systems. Practical applications of these findings range from technical diagnostics to the development of new arc quenching technologies.

Future research by the authors’ team will focus on the photographic analysis of discharges in the presence of other residual gases and with different contact materials. Conducting a comparative analysis will allow conclusions to be drawn about the influence of these factors on discharge development.

## Figures and Tables

**Table 1:** Characteristics of type II cathode spots [1].

Contact Condition	Cleaned by Arcing
Velocity	1–100 ms^–1^
Spot current	30–300 A
Crater radius	40–100 μm
Erosion rate	10–100 µgC^−1^

**Table 2:** Point erosion rates of cathodes for metals used in vacuum interrupter contacts [1].

Material	Erosion Rate
	µgC^−1^
Ag	140, 150
Cu	115, 130
Cr	22–40
W	55, 62, 64

**Table 3:** Effect of overlapping plasma plumes from cathode points with increasing current [1].

View of a Scattered Vacuum Arc	Description of the Burning Vacuum Arc Between the Contacts of the Vacuum Interrupter
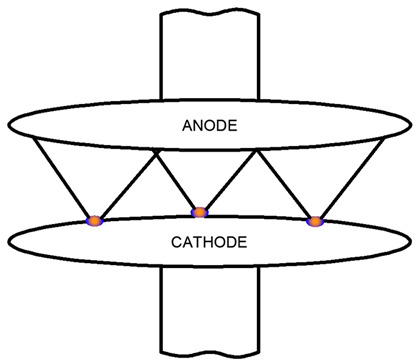	Low-current, distributed vacuum arc. Slight overlap of plasma clouds over cathode points.
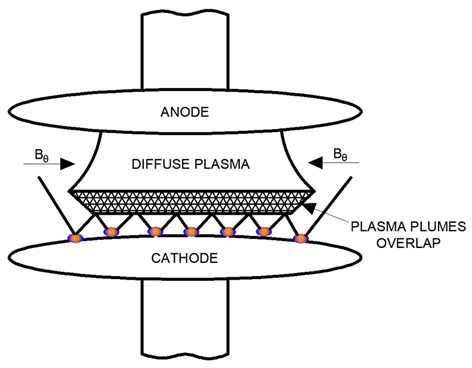	Diffuse vacuum arc with higher current value. Significant overlap of plasma clouds over cathode points. Visible compression of the arc column.

**Table 4:** Measurement parameters set for vacuum arc testing using the photographic method.

Parameter	Value
Resolution	512 × 384
Sampling frequency	33,000 frames/s
Exposure time	10 µs
Pressure	4.00 × 10^−3^ Pa ÷ 4.00 × 10^1^ Pa
Power supply parameters	10 A, 230 V

## Data Availability

The original contributions presented in the study are included in the article; further inquiries can be directed to the corresponding author.
